# Analysis of development and evolution rules of civil aviation in China based on life cycle theory

**DOI:** 10.1371/journal.pone.0212338

**Published:** 2019-02-20

**Authors:** Yifei Zhao, Junqiang Wan

**Affiliations:** National Key Laboratory of ATC Safety Technology, Civil Aviation University of China, Tianjin, China; King Abdulaziz University, SAUDI ARABIA

## Abstract

The development of CAAC began in the early days of 1949. From a comparatively less popular means of transport to the world's second largest by volume, this means of transport has undergone major and minor changes in the last 70 years. It is not known whether there are significant laws in the process of development. For this reason, we analyze the statistical indicators of the development of civil aviation transport and select representative indicators, namely, the total turnover of transport, the number of routes, the number of aircraft, the number of transport aircraft, and the number of domestic city connections. At the same time, the life cycle theory is introduced, and the typical growth curve model is used to fit the data. It is found that the evolution life cycle of civil aviation in China can be divided into three stages: the first life cycle stage from 1950 to 1981, the second from 1982 to 2003, and the third from 2004 to 2017. Each life cycle follows the growth characteristics of occurrence, growth and maturity, and each life cycle has a time range of approximately 30 years. At present, China's civil aviation industry is in the period of rapid growth in the third life cycle. This industry is expected to reach maturity in approximately 2026 and then to begin to grow slowly. Relevant departments can adopt corresponding development strategies to guide the smooth development of civil aviation in accordance with the growth law of the development and evolution life cycle of civil aviation in China.

## 1 Introduction

Civil aviation refers to the use of various types of aircraft engaged in all aviation activities other than national defense, police and customs. Civil aviation is primarily divided into two parts: public air transport and general aviation transportation.

**Fig 1:**
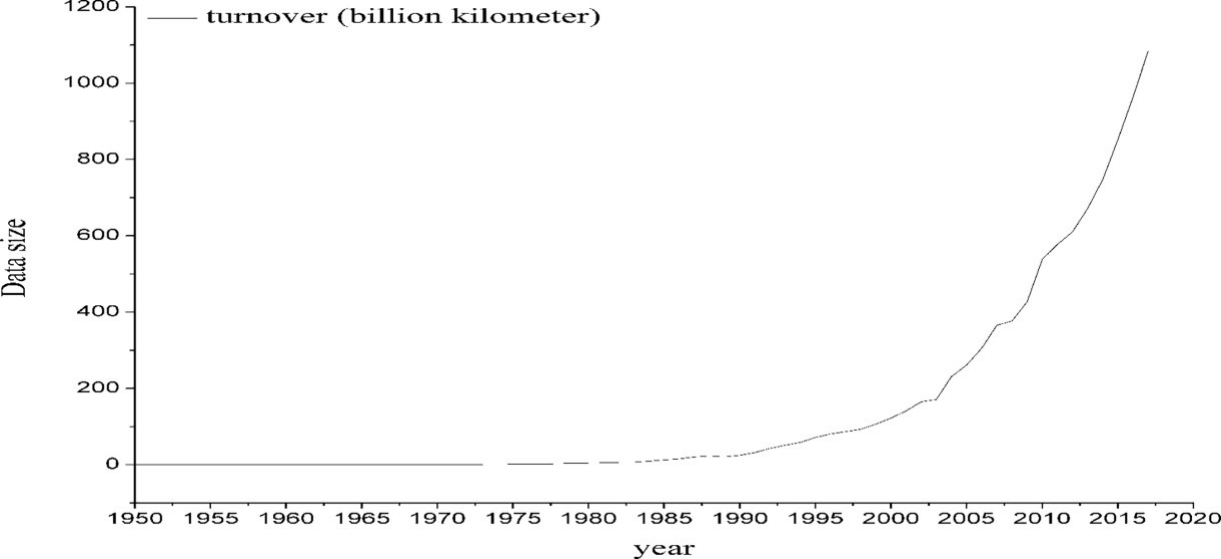
China civil aviation passenger traffic turnover growth chart from 1950 to 2017.

The origin of China's civil air transport can be traced back to the 1920s. China Airlines was founded in 1929, and then Eurasia Airlines, a Sino-German joint venture, was established (and was reorganized as the Central Airlines in 1943). However, at that time, the level of social and economic development was relatively low, and the development of air transport was also notably slow. By 1950, the total turnover of transport was only 1.57 million tons of kilometers, passenger traffic was 10.4 million people, the number of domestic and international routes was only 12, the number of transport aircraft was 30, and the number of domestic and international city connections was only 8. By 1978, the total turnover of transport reached 29.86 million tons of kilometers, passenger transport capacity reached 2.39 million people, the number of domestic and international routes reached only 162, aircraft reached 508 (transport aircraft reached 144), and domestic and international city connections reached only 93. From 1978 to 1997, the total transport turnover and passenger turnover maintained an average annual growth rate of over 19%, considerably higher than the 6% level in the same period in the rest of the world. In 1997, the total turnover of China's civil aviation transportation was 8.67 billion tons kilometers, accounting for 2.4% of the world's total, only 7.1% of the total turnover of the United States, ranking 10th in the world. China's passenger turnover of 56.297 million, accounting for only 2.8% of the world's total, equivalent to 7.4% of U.S. passenger turnover, is still far below Australia's level. In 2002, China's total air transport turnover was 16.49 billion tons kilometers, ranking fifth in the world, and its passenger turnover was 85.94 million, ranking fourth in the world. The annual growth rate of total turnover was 16.8%, which was 15.2 percentage points higher than the world average; the growth rate of passenger turnover was 16.3%, which was 15.9 percentage points higher than the world average. In 2005, China's total turnover reached 26 billion 130 million tons, ranking second in the world [1]. By the end of 2016, China's air transport scale has been ranked second in the world for 12 consecutive years, second only to the United States. The proportion of civil aviation passenger turnover in the comprehensive transport system reached 26.8%, 11.7 percentage points higher than in 2012, and the airport network with the largest population coverage and the fastest growing speed in the world was built [2]. The passenger flow volume growth map from 1950 to 2017 is shown in Fig 1.

Along with the growth of civil aviation transportation, there are several major reforms in the development process. The first stage was the early founding of New China, i.e., the construction of China's civil aviation management system (before 1980). From 1949 to 1979, the government completely controlled the civil aviation industry and implemented a monopoly. The government plays three roles in the operation of civil aviation: organizer, decision-maker and manager. The army holds complete and unified leadership over the civil aviation industry.

The second stage was the reform with the theme of "conversion of military to civilian and entrepreneurship" (1980–1987). Comrade Deng Xiaoping put stated in 1980 that "civil aviation must be handled via entrepreneurship," which began the reform of civil aviation administrative system with the core of entrepreneurship and the means of market-oriented reform. Although the civil aviation industry is separated from the military system and changed into a directly affiliated institution under the State Council and is under the management of enterprises, the civil aviation bureau directly operates the air transport business while assuming the responsibility of the government departments, and the state monopoly operation mode under the civil aviation industry government-enterprise integration system has not changed.

The third stage was the "separation of government and enterprise and separation of airport and airline" as the main direction of reform (1987–2002). In 1987, the State Council approved the Civil Aviation Administration of China's "Civil Aviation System Management System Reform Program and Implementation Steps Report", which began to take the separation of airlines and airports as the main content of the reform. As a special department of the State Council in charge of civil aviation, the Civil Aviation Administration exercises the functions of the government, assumes the responsibilities of industry supervision, and does not directly direct the command and operation of aviation business. In the context of reform and opening up, the aviation industry has been increasingly involved in international market exchanges, cooperation and competition. Airports and airlines operate according to the enterprise system.

The fourth stage was the reform based on "separation of government and capital and localization of airports" (2002–2007). In 2002, the State Council approved the "Civil Aviation System Reform Plan" and initiated reform based on "separation of government and capital and localization of airports". The reform plan clearly states that "the CAAC shall assume the functions of civil aviation safety management, market management, air traffic management, macrocontrol and external relations, stop acting as the owner of state-owned assets of major group companies, and transfer them to the state-owned assets management department of the State Council. The civil aviation administrative bureaus of provinces, districts and municipalities shall separate the government from the enterprises and set up airport administrative organs and aviation safety supervision offices. To implement the reform of airport localization, 90 airports in addition to the Capital International Airport Group Corporation and the Tibet Airport will be transferred to local government administration.

The fifth stage is the reform with the key of "the reform of large department system and the acceleration of the comprehensive transportation system" (2008-): To make full use of the interconnection, overall superiority and combination efficiency of various modes of transportation and to form a comprehensive transportation system, the Ministry of Communications and the General Civil Aviation Administration of China are to be established in accordance with the Institutional Reform Plan of the State Council. The responsibilities of the Bureau and other institutions shall be integrated into the Ministry of Transport; the State Civil Aviation Administration shall be established and administered by the Ministry of Transport; and the Ministry of Communications and the General Administration of Civil Aviation of China shall no longer be retained. Since then, the civil aviation administrative system of "two levels of government and three levels of management" has been formed.

Life cycle generally refers to the periodic change and development law of various objective things in the evolution process of nature and human society. The evolution of world aviation has gone through nearly 120 years. Gilbert [3], the first generation of American air traffic controllers and the father of American air traffic management, divides the development of civil aviation air traffic management into four stages: radio air traffic management (before 1936), radar air traffic management (before 1950), automated air traffic management (1960s), and future air traffic management (1970s). Kraus [4], an American aviation historian, has analyzed in detail the major changes that have taken place in the air traffic management system in the United States in the decade from the mid-1930s to the mid-1940s. In the same year, Cistone [5], founder of NextGen, wrote a comprehensive review of the development of air traffic management from 1903 to 2000 in a 10-year period. The National Air Traffic Controllers Association (NATCA) [6] brings together opinions and divides air traffic control into seven stages: The dawn of aviation (1903–1925), procedure control (1926–1935), radar control (1936–1957), jet age (1958–1977), the turbulent period of aviation system (1978–1993), coexistence of technological progress and challenges (1994–2001), and future development (2002-). Research shows that the development and development of civil aviation in the United States conforms to the evolution rule of life cycle. At present, China's civil aviation is showing a trend of rapid development, and a large number of new aircraft and equipment are being added as part of the planning and construction. However, whether the future of civil aviation in China will show similar evolutionary laws to those of Western countries and whether it will face the same dilemma as the development of civil aviation in the United States deserves our deep consideration.

At present, the main viewpoints on the development and evolution of civil aviation in China are as follows: Li [7] divided the reform and development of China's civil aviation transport industry into four stages from the perspective of a management system, points out the problems existing in the current system reform, and gives his own suggestions. Chen [8] analyzed the impact of changes in system and demand on China's air transport industry from the perspective of the evolving environment of China's air transport industry and analyzed the existing problems in the evolution and development of China's air transport industry. Based on the theory of evolutionary economics, an evolving model of air transport industry was constructed, and some suggestions were made for the development of China's air transport industry [8]. Shen [9] analyzed the reform process of China's civil aviation system from the perspective of market-oriented reform and prospected the further market-oriented reform of China's civil aviation management system under the new normal economy.

The above studies are all from the perspective of civil aviation transport system reform and economic development to analyze the development and evolution of China's civil aviation without quantitative data analysis of the development of China's civil aviation transport industry. Quantitative research starts with determining quantifiable development indicators, uses data mining methods to analyze annual differences, and then determines the development stage. This study attempts to divide the stage of development and evolution of civil aviation in China from the perspective of quantitative development indicators.

Life cycle, as a concept of biology, has been widely used in many fields, such as environment, economy, technology and society. Life cycle generally refers to the periodic changes of objective things in the process of development and evolution. Chiu and Yen [10] introduced the theory of organizational life cycle to analyze the port reform and development in Taiwan. Cox et al. [11] carried out air traffic life cycle assessment for 72 common aircraft types with different flight distances. Coelho et al. [12] used life cycle theory in the evaluation of dietary environment. Zhang et al. [13] used life cycle theory in energy efficiency and environmental impact. Islam et al. [14] used life cycle theory to evaluate residential roofs and floors.

The growth curve is a kind of curve model describing the variable of the object of study changes with time and presenting a certain biological change law. The growth curve usually goes through three stages: occurrence, growth and maturity. The growth curve’s theory can quantitatively describe and study the life cycle evolution law of the object of study [15].

Only on the basis of fully understanding the development process of civil aviation in China, through basic and frontier research, can we correctly grasp the law of the development and evolution of civil aviation in China to correctly guide the future development of China's civil aviation and promote the rapid and efficient transformation and development of the civil aviation industry. This study uses life cycle theory to identify the evolutionary life cycle of the development of civil aviation in China from 1949 to 2017 and discusses the evolutionary growth law of civil aviation transportation in the past 70 years. At the same time, the study predicts the development trend of civil aviation transportation to provide a reference for managers to guide the planning of civil aviation transportation development.

## 2 Technical route analysis

### 2.1 Data acquisition

This paper focuses on the collection of data released by the Civil Aviation Administration of China from 1949 to 2017. There are two sources of data; one is "Statistical data on Civil Aviation of China", and the other is "The Chronicle of China’s Civil Aviation (1949–2010)".

"Statistical data on Civil Aviation of China" is responsible for data collection, collation and publication by the Civil Aviation Administration of China and is published annually in the form of publishing books. The book includes six categories of statistical indicators: resource profile, air transport development indicators, general aviation development indicators, efficiency indicators, aviation safety and service quality evaluation indicators, and historical development indicators. "Resource profile" includes all kinds of resources owned by the entire industry, including the number of routes, city connections, aircraft, airports, enterprises and personnel; "air transport development indicators" includes the transport indicators of the entire industry, airlines and airports. "General aviation development indicators" includes industry-wide general aviation indicators and subproject operations. "Efficiency indicators" include financial revenue, flight efficiency and aircraft utilization of civil aviation enterprises. "Aviation safety and service quality evaluation indicators" includes flight safety indicators, airline flight normal rate and airport release normal rate indicators and passenger complaints. "Historical development indicators" includes the historical data of air transport, general aviation, airports and other transport indicators of the entire industry.

"The Chronicle of China’s Civil Aviation (1949–2010)" is the history of civil aviation development compiled and issued by the Civil Aviation Administration of China. The statistical data provided in the book are basically consistent with those in "Statistical data on Civil Aviation of China". This study mainly uses the statistics before 2010 as a supplement to "Statistical data on Civil Aviation of China". In addition, "The Chronicle of China’s Civil Aviation (1949–2010)" also provides information about the development of civil aviation, the leaders of civil aviation bureaus and other aspects, which can be compared with the quantitative analysis done in this study.

From the "Statistical data on Civil Aviation of China" and "The Chronicle of China’s Civil Aviation (1949–2010)", the data collected in this study include total transport turnover, passenger turnover, cargo and mail turnover, aircraft number, route number, and route mileage. Total transport turnover, passenger turnover, cargo and mail turnover, and aircraft number are used to represent the development needs of air transport, while route number and route mileage are used to represent the level of infrastructure construction. It should be noted that the number of airports in China is not an important parameter in the two documents, so this study uses the number of domestic navigable cities with a very close connotation to replace it. This finding is observed because by the end of 2018, except for Shanghai, there is only one airport in every city in China, and the correlation between the two is very high.

### 2.2 Data analysis and processing

It is noteworthy that as the development of civil aviation will be reflected in many aspects, the above selected transport turnover, passenger turnover, cargo and mail turnover, number of routes, route mileage, number of aircraft, number of city connections and other quantitative indicators are likely to be associated. If the highly correlated data are considered at the same time, the impact of other indicators on the final analysis results will be weakened. Therefore, this study will introduce the correlation test link, calculate the Pearson correlation coefficients (PCC) of two indicators, and make a choice between the high correlation indicators.

Pearson correlation coefficient is a measure of linear dependence between two random variables (real valued vectors). Historically, this coefficient is the first formal measure of relevance, and it remains one of the most widely used relational measures. Pearson correlation coefficients of two variables *x* and *y* are formally defined as the product of the covariance of two variables divided by their standard deviation (as a standardized factor), and can be defined equivalently by [16,17]

$$\begin{array}{l} r_{xy}=\frac{\sum \left(x_{i}-\bar{x})(y_{i}-\bar{y}\right)}{\sqrt{\sum {\left(x_{i}-\bar{x}\right)}^{2}}\sqrt{\sum {\left(y_{i}-\bar{y}\right)}^{2}}} \end{array}$$ (1)

In this equation, $\bar{x}=\frac{1}{n}\sum_{i=1}^{n}x_{i}$ represents the mean of *x*, and $\bar{y}=\frac{1}{n}\sum_{i=1}^{n}y_{i}$ represents the mean of *y*. The coefficient *r*_*xy*_ ranges from −1 to 1, and it is invariant to the linear transformation of any variable.

PCC gives an indication of the strength of the linear relationship between two random variables *x* and *y*. If the variables are directly related, the symbols of the correlation coefficients are positive. If the variables are inversely related, the symbols of the correlation coefficients are negative. If *r*_*xy*_ = 0, then *x* and *y* are called irrelevant. The closer the value of *r*_*xy*_ is to 1, the closer the measure is to the linear relationship.

According to the theory of correlation analysis, when 0.5<|*r*_*xy*_|≤0.8, it is considered that the two indicators are significantly correlated; when 0.8<|*r*_*xy*_|≤1, it is considered that the two indicators are highly correlated; when |*r*_*xy*_| = 1, it is considered that the two indicators are completely correlated. This study assumes that when *r*_*xy*_≥0.85, the two indicators have good correlation.

### 2.3 Life cycle partition model

After completing the correlation test, there will still be a number of quantitative indicators; therefore, the data should be normalized. After normalization, the indexes, such as the total turnover and the number of aircraft, constitute a comprehensive data. The next step is to use the growth curve theory to classify the development of China's civil air transport.

#### 2.3.1 Standardization of data

Because the physical dimensions of the selected indicators are different, it is impossible to make a comprehensive score on the development indicators of civil aviation transportation. Therefore, this study adopts the centralized dimensionless method to deal with the dimensionless classification indicators. The calculation formula is as follows [18].

$$x^{'}=[(x-\bar{x})/S]\cdot c+d$$ (2)

In this equation, $\bar{x}$ denotes the sample mean of the index, *S* denotes the sample standard deviation of the index, and *c* and *d* denote the scaling and translation of the values after dimensionless transformation, respectively.

#### 2.3.2 Calculation of index weight

Before data normalization, we need to weight each index. According to Chebyshev inequality theorem, we can see [19,20]

Suppose the mean of random variable *X* is *E*(*X*) = *μ*, and the variance is *D*(*X*) = *σ*^2^. For ∀:*ε*∈N+, there is *P*{|*X*−*μ*|≥*ε*}≤*σ*^2^/*ε*^2^.

For the index *X*_*i*_, assuming that $m_{i}=\underset{1\leq j\leq m}{\min}(X_{ij})\leq X_{i}\leq \underset{1\leq j\leq m}{\max}(X_{ij})=M_{i}$ is established, there is:

$$p_{i}=P\left\{m_{i}\leq X_{i}\leq M_{i}\right\}=P\left\{m_{i}-\mu_{i}\leq X_{i}-\mu_{i}\leq M_{i}-\mu_{i}\right\}$$ (3)

Obviously, *m*_*i*_≤*μ*_*i*_≤*M*_*i*_ is feasible, set *ε*_1_ = min{|*M*_*i*_−*μ*_*i*_|,|*m*_*i*_−*μ*_*i*_|}, there is

$$\left\{m_{i}-\mu_{i}\leq X_{i}-\mu_{i}\leq M_{i}-\mu_{i}\right\}⊃\left\{-\varepsilon_{i}\leq X_{i}-\mu_{i}\leq \varepsilon_{i}\right\}⊃\left\{-\varepsilon_{i}<X_{i}-\mu_{i}<\varepsilon_{i}\right\}$$ (4)

And then

$$P\left\{m_{i}-\mu_{i}\leq X_{i}-\mu_{i}\leq M_{i}-\mu_{i}\right\}\geq P\left\{-\varepsilon_{i}\leq X_{i}-\mu_{i}\leq \varepsilon_{i}\right\}\geq P\left\{-\varepsilon_{i}<X_{i}-\mu_{i}<\varepsilon_{i}\right\}$$ (5)

Thus, we can get:

$$p_{i}=P\left\{m_{i}\leq X_{i}\leq M_{i}\right\}\geq P\left\{\left|X_{i}-\mu_{i}\right|<\varepsilon_{i}\right\}\geq 1-\sigma_{i}^{2}/\varepsilon_{i}^{2}$$ (6)

We can set *r*_*i*_ = *σ*_*i*_/*ε*_*i*_: therefore, from Eq (6) it can be seen that the size of *r*_*i*_ reflects the lower limit of the probability of the range of index *X*_*i*_. The smaller the value of *r*_*i*_, the greater the lower limit of *p*_*i*_, and the greater the contribution of index *X*_*i*_ to the scheme.

For equation $e_{i}=\min(p_{i})=1-r_{i}^{2}$, the greater the value of *e*_*i*_, the greater the effect of index *X*_*i*_, that is, the index has been given a greater weight, so the weight of index *X*_*i*_ can be expressed as

$$W_{i}=\frac{e_{i}}{\sum_{i=1}^{n}e_{i}}(i=1,2,3,\ldots ,n)$$ (7)

#### 2.3.3 Growth curve theory

**Fig 2:**
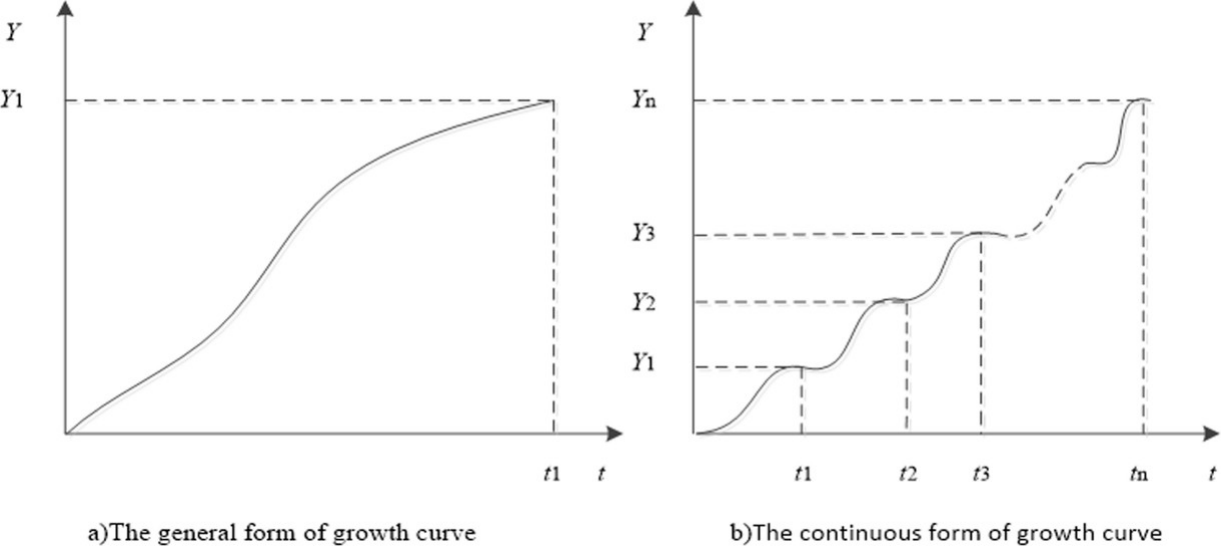
Growth form of growth curve.

Using mathematical methods to fit curves, it is possible to use linear or exponential functions sometimes in line with the law of growth, but in most cases, we should start from the S-shaped growth curve. The growth curve can be divided into a single growth curve and a continuous growth curve according to the number of growth times *n* [21]. As shown in Fig 2.

#### 2.3.4 Growth curve model based on adjusting goodness of fit

For the current curve fitting, the goodness of fit *R*^2^ between the analysis curve and the model curve is used to discriminate. The goodness of fit can be used to characterize the fitting degree between the actual collected data and the fitted equation.

Suppose that there are *n* values to be fitted for variables *x*_*i*_ and *y*_*i*_, and the mean values are $\bar{x}$ and $\bar{y}$, respectively. The total square sum of the samples is *SST*, the sum of squares of errors is *SSE*, and the sum of squares of regression is *SSR*. Then, there is *SST* = *SSR*+*SSE* [22]

Goodness of fit *R*^2^ can be expressed as

$$R^{2}=\frac{SSR}{SST}=1-\frac{SSE}{SST}$$ (8)

At the same time, *R*^2^ can be expressed as

$$R^{2}=\frac{SSR}{SST}=\frac{[\sum x_{i}y_{i}-n\bar{x}\bar{y}]/\sum {\left(x_{i}-\bar{x}\right)}^{2}}{\sum \left(y_{i}^{2}-n{\bar{y}}^{2}\right)}=\frac{[\frac{\sum x_{i}y_{i}-n\bar{x}\bar{y}}{n-1}{]}^{2}}{\frac{\sum {\left(y_{i}-\bar{y}\right)}^{2}}{n-1}\cdot \frac{\sum {\left(x_{i}-\bar{x}\right)}^{2}}{n-1}}=\frac{S_{XY}^{2}}{S_{X}^{2}S_{Y}^{2}}=\gamma_{XY}^{2}$$ (9)

In applications, you will find that if you add an explanatory variable in the model, *R*^2^ will become larger. However, the magnitude of *R*^2^ should not be related to the increase of explanatory variables. The main reason for this is that *SST* and *SSE* are not taken into account in the calculation; therefore, the adjusted goodness of fit can be defined as

$${\bar{R}}^{2}=1-\frac{SSE/(n-2)}{SST/(n-1)}$$ (10)

Compared with goodness of fit, adjusting goodness of fit is better because it eliminates the influence of the number of variables. Therefore, when judging the life cycle of the evolution and development of China's civil aviation transportation, we should choose to adjust the goodness of fit for analysis and judgment.

## 3 Determining quantifiable indicators

### 3.1 Selection of representative indicators

The data in this study are the air transport statistics of CAAC from 1950 to 2017, which are collected from "Statistical data on Civil Aviation of China" and "The Chronicle of China’s Civil Aviation (1949–2010)". There are 10 indicators. They are: total transport turnover, passenger traffic volume, passenger turnover volume, cargo and mail traffic, cargo turnover, number of routes, route mileage, number of Aircraft, transport aircraft, number of city connections. Then, pearson's principle of relational analysis is used to screen indicators to ensure scientific analysis [1,23–29].

From left to right, V_1_, V_2_, V_3_ …,V_10_ are used to represent 10 indexes in Table 1, respectively. Then, the correlation of index data is analyzed. The result is presented in Table 2.

**Table 1:** Analytical indicators of CAAC transport development.

Year	Total transport turnover (100 million tons kilometers)	Passenger traffic volume (10000 passengers)	Passenger turnover volume (10000 passenger-kilometer)	Cargo and mail traffic (tons)	Cargo turnover (10,000 tons of kilometers)	Number of routes	Route mileage (km) unduplicated	Number of Aircraft(frames)	Transport aircraft(frames)	Number of city connections(pieces)
1950	0.0157	1.04	978	767	82	12	11369	30	30	7
1951	0.0349	2.39	2261	1670	168	11	11249	59	59	9
1952	0.0435	2.2	2409	2047	243	10	13123	45	45	12
1953	0.0712	2.8	3322	3608	447	13	13950	47	47	9
1954	0.0969	4.2	5017	4734	560	11	15243	54	49	23
…	…	…	…	…	…	…	…	…	…	…
2013	671.7	35397	56567596	5612526	1702918	2876	4106000	3827	2173	188
2014	748.1	39195	63341903	5940988	1877715	3142	4637214	4168	2370	198
2015	851.65	43618	72825500	6293000	2080700	3326	5317000	4554	2650	204
2016	962.51	48796	83781300	6680000	2224500	3794	6348000	5046	2950	214
2017	1083.1	55156.8	95128000	7058000	2435000	3872	7300000	6037	3261	224

**Table 2:** Results of correlation analysis of index data.

	1950–1959	1960–1969	1970–1979	1980–1989	1990–1999	2000–2009	2010–2017
V_1_-V_2_	0.991776	0.9196164	0.9974423	0.9957955	0.9816459	0.9979653	0.9977141
V_1_-V_3_	0.9782808	0.8647163	0.9989072	0.9994842	0.9899032	0.9991268	0.9986507
V_1_-V_4_	0.9788896	0.8960241	0.9750378	0.9979434	0.9934546	0.9931808	0.9739828
V_1_-V_5_	0.9886885	0.9407915	0.9918747	0.997733	0.9806225	0.9912187	0.9510315
V_1_-V_6_	↓0.6328482	↓0.3274516	0.9741432	0.9809629	0.9873792	0.9748251	0.9629836
…	…	…	…	…	…	…	…
V_7_-V_9_	↓0.8361141	↓0.7583565	↓0.7936937	↓0.7467797	0.9898296	0.9433809	0.9857989
V_7_-V_10_	↓0.7840976	↓0.4546845	↓0.7694287	0.8697353	0.9368333	↓0.8456327	0.9898989
V_8_-V_9_	↓0.8186285	↓0.671981	0.9604186	0.8932437	0.9924856	0.9979305	0.9942507
V_8_-V_10_	↓0.5695367	↓0.6761974	0.9270556	↓0.8374298	0.9762593	0.9656385	0.9853799
V_9_-V_10_	↓0.7048356	↓0.5479263	0.952136	↓0.8433377	0.9587742	0.961422	0.988633

Note: the data with "↓" represent less than 0.85, and the correlation is not good.

According to the correlation analysis in Table 2, we can see that the correlation between V_1_ and V_2_, V_3_, V_4_, V_5_, V_7_ has been good; therefore, one of them can be chosen. Then, considering the inherent characteristics of air transport, five indicators are selected to reflect the development process of China's civil aviation transport, namely, the total transport turnover, the number of routes, the number of aircraft, the number of transport aircraft, and the number of city connections (i.e., V_1_, V_6_, V_8_, V_9_, V_10_).

At the same time, in order to simplify the calculation and ensure the accuracy of the results, this study selects the representative data of China's civil aviation transportation in the past 10 years (2009–2017) to calculate the weight of each index, and the results are listed in Table 3.

**Table 3:** Weight of representative index.

index	V_1_	V_6_	V_8_	V_9_	V_10_
weight	0.19651	0.18839	0.16000	0.19720	0.25790

### 3.2 Dimensionless processing and normalization of indicators

According to the index data in Table 1 and the dimensionless processing formula introduced above, the evaluation index data are dimensionlessly processed. To facilitate analysis, this study selects *c* = 1, *d* = 1. The dimensionless processing results of the five representative indicators are presented in Table 4.

**Table 4:** Dimensionless processing score of CAAC air transport assessment indicators.

Year	Total transport turnover	Number of routes	Number of aircraft	Number of Transport aircraft	Number of city connections
1950	-0.5428	-0.7308	-0.7792	-0.6510	-1.6259
1951	-0.5427	-0.7319	-0.7563	-0.6129	-1.5886
1952	-0.5427	-0.7329	-0.7673	-0.6313	-1.5327
1953	-0.5426	-0.7298	-0.7658	-0.6287	-1.5886
1954	-0.5425	-0.7319	-0.7602	-0.6260	-1.3277
…	…	…	…	…	…
2013	2.0944	2.2221	2.2276	2.1684	1.7470
2014	2.3944	2.4964	2.4976	2.4276	1.9334
2015	2.8009	2.6861	2.8033	2.7960	2.0452
2016	3.2362	3.1687	3.1929	3.1907	2.2315
2017	3.7097	3.2491	3.9777	3.5999	2.4179

**Fig 3:**
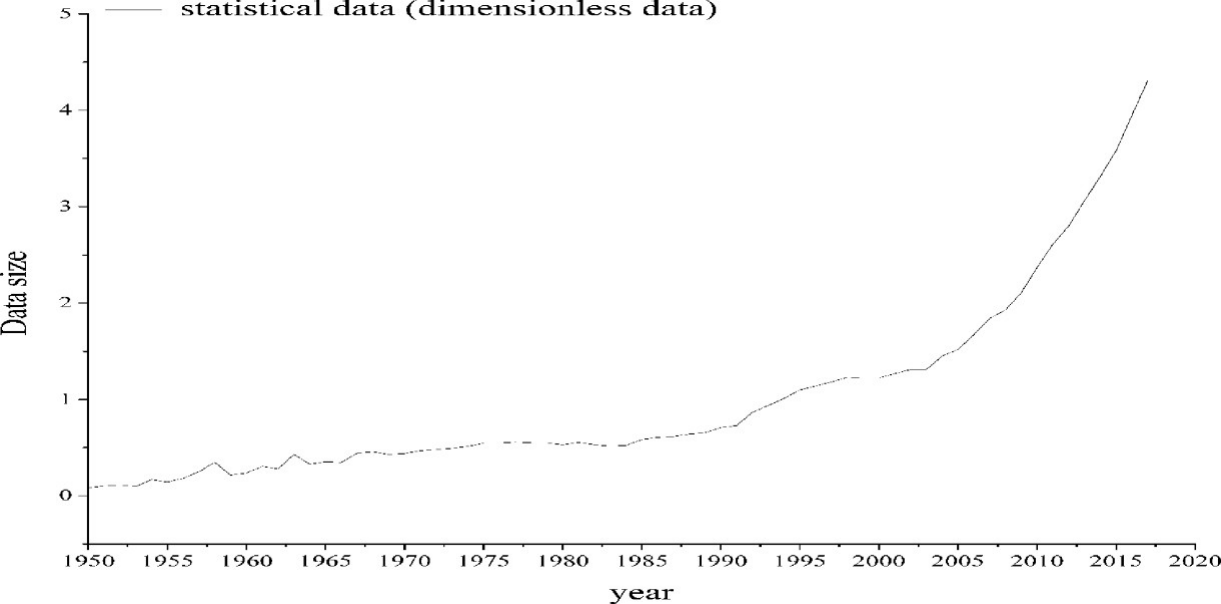
Statistical data curve of china civil aviation development level.

After the calculation of the weights in the previous section, the dimensionless data in Table 4 are weighted and normalized to obtain the comprehensive score value of the development level of civil aviation transportation in China, and a graph is plotted, as shown in Fig 3.

## 4 Evolution of China's civil aviation transportation life cycle

According to the trend of the curve in Fig 3, the evolution curve of civil aviation transportation in China is a kind of nonlinear infinite growth curve. The evolution law and life cycle of the curve can be described by using the nonlinear growth curve function. The evolution data can be fitted by using the logistic function model, and the fitting results diverge, which are clearly inconsistent with each other. It is assumed that the evolution of China's civil aviation transportation conforms to the basic characteristics of the continuous growth curve.

**Fig 4:**
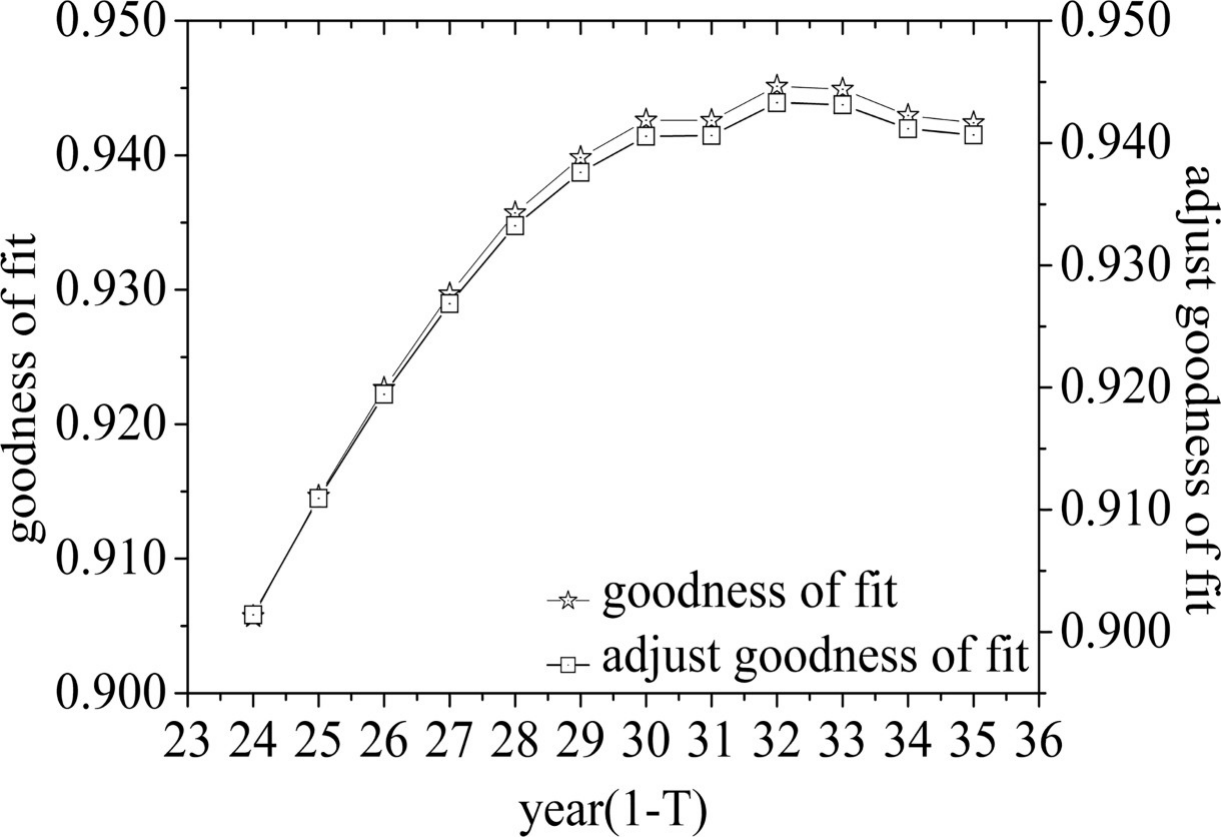
Goodness of fit and adjustment of goodness of fit curve of double Y-axis(the first life cycle).

**Fig 5:**
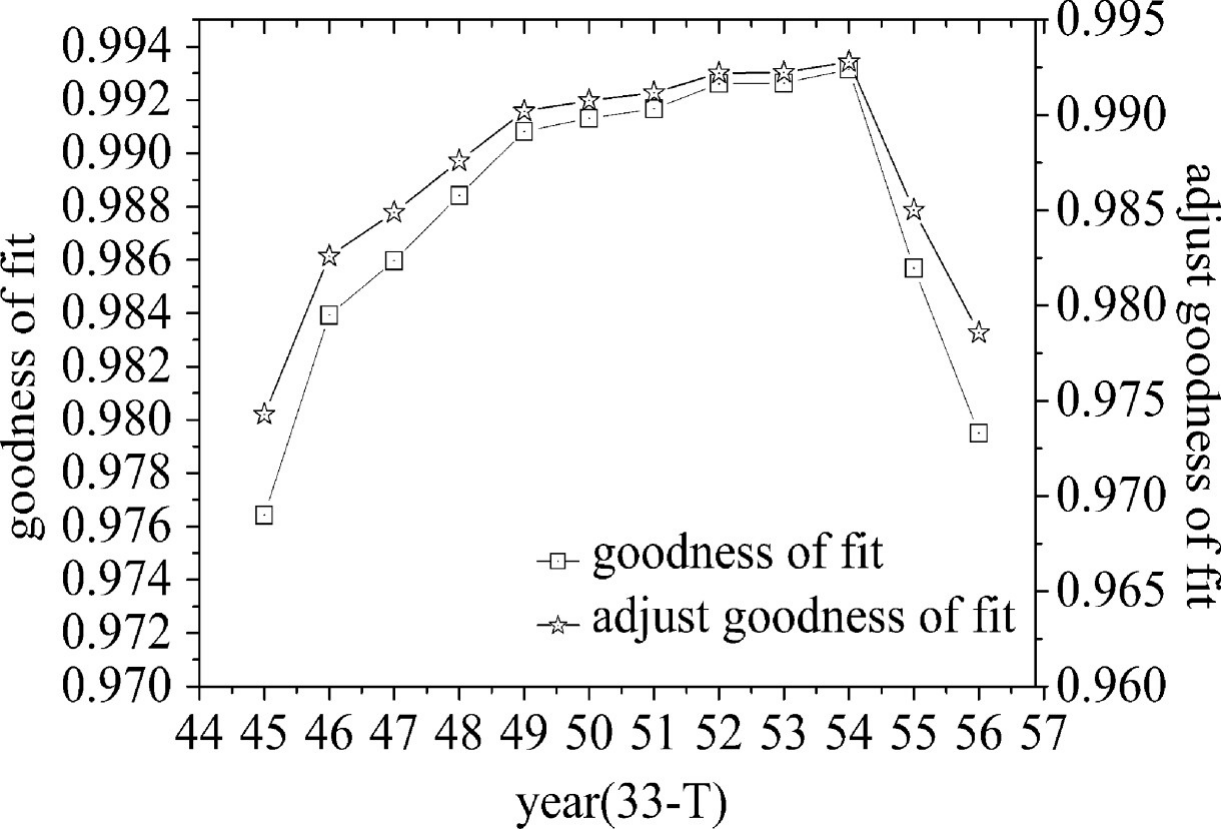
Goodness of fit and adjustment of goodness of fit curve of double Y-axis(the second life cycle).

**Fig 6:**
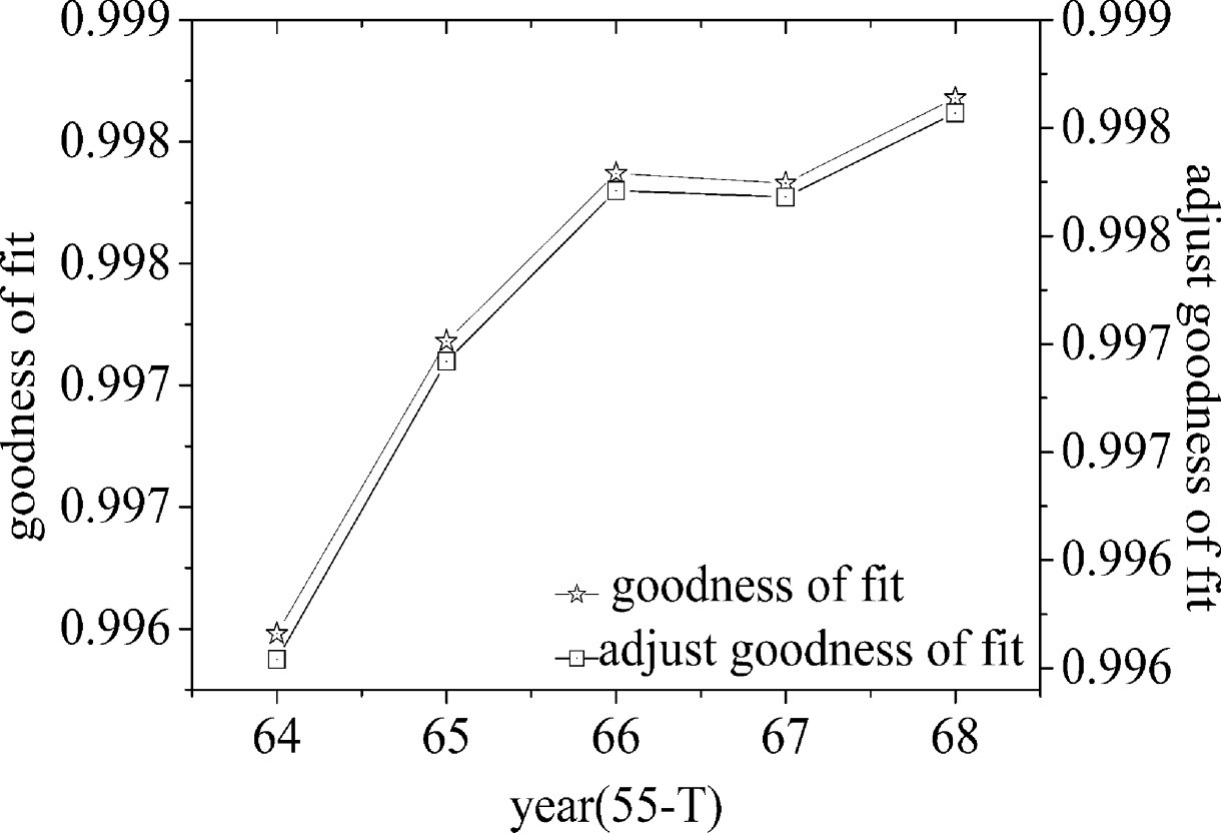
Goodness of fit and adjustment of goodness of fit curve of double Y-axis(the third life cycle).

The validity of goodness-of-fit usually requires (number of independent variables/samples≥(1/10)); therefore, the first 10 years from 1950 to 1959 are used as the fitting starting point of the first life cycle, and the adjusted goodness-of-fit of different time periods are calculated. Then, the maximum membership principle is used to identify and judge the first life cycle. At the same time, in order to avoid the large value of the random variable *x* and affect the accuracy of the parameters, the random variable is adjusted to the *t t* year, that is, 1950 is the first year, extending to the 68th year of 2017, a total of 68 time nodes. By calculating and analyzing and removing the fitting divergence points, the fitting goodness of each life cycle and the double Y-axis line chart of adjusting the fitting goodness can be obtained, as shown in Fig 4, Fig 5 and Fig 6.

Through the analysis of Fig 4, it can be seen that from the 24th node, the adjustment goodness-of-fit has been on the rise, reached the maximum at the 32nd node, and then decreased with the passage of time. Fig 5 shows that with the passage of time, the adjusted goodness-of-fit reaches its maximum at the 54th time node and then shows a downward trend, that is, the second life cycle period is 1982–2003. Fig 6 shows that the adjusted goodness of fit has been on the rise over time, that is, the third life cycle period is from 2004 to 2017.

## 5 Life cycle growth curve fitting and parameter estimation

### 5.1 Recognition of growth curve model

Generally, *q*(2≤*q*≤5) alternative growth curve models are selected to fit the original data, and the curve characteristics of the alternative models are consistent with the growth characteristics of the actual curves [22].

Because the growth curve model has many forms, and each model has different characteristics, in the process of fitting the evolution and development life cycle of China's civil aviation transportation, we should use a variety of fitting models, and then choose the model with the highest fitting degree as the fitting model. If the fitting degree of logistic is optimal, then the life cycle division of civil aviation development and evolution is convincing.

**Fig 7:**
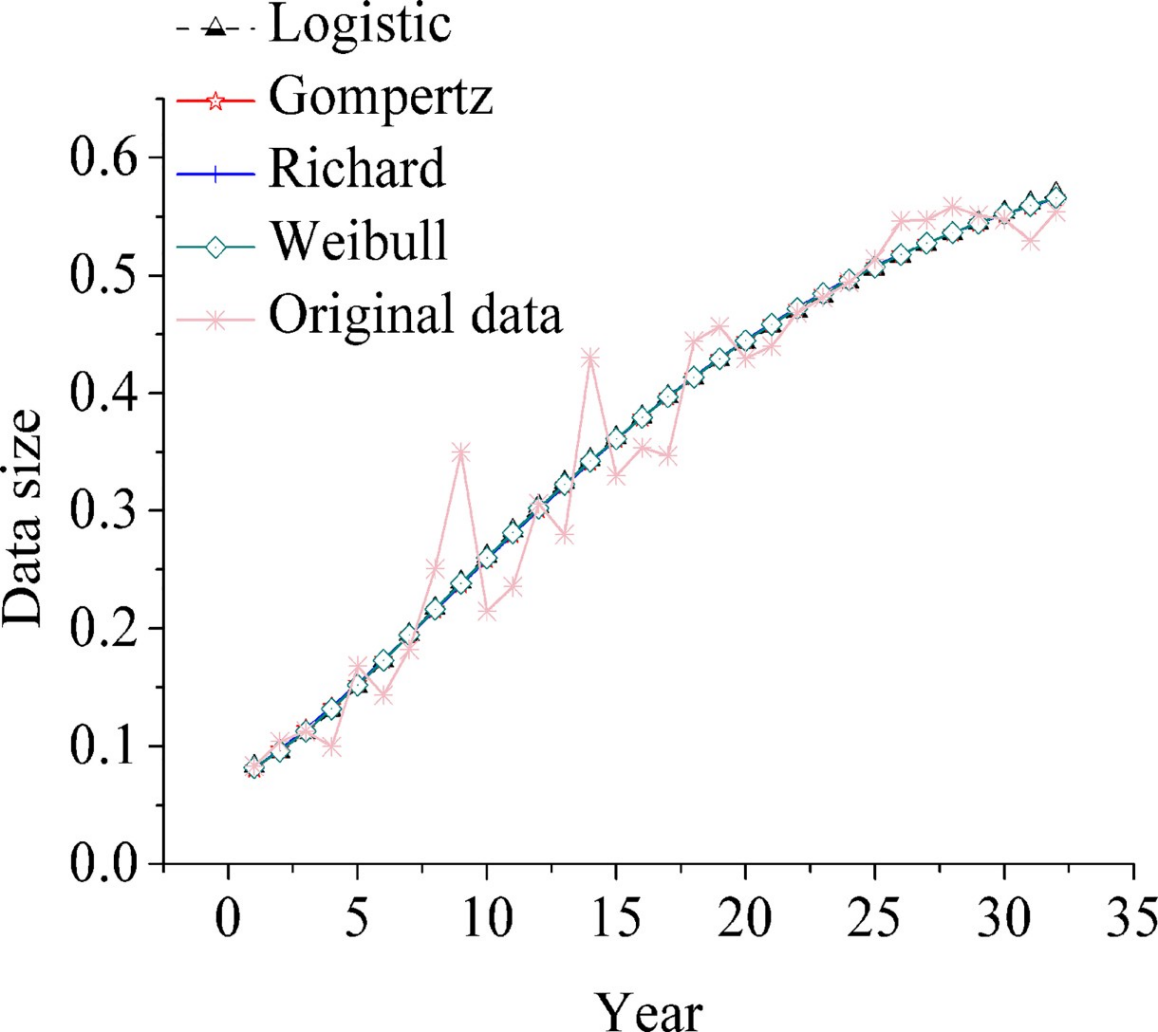
Comparison of four growth curve fitting curves in three life cycle (the first life cycle).

**Fig 8:**
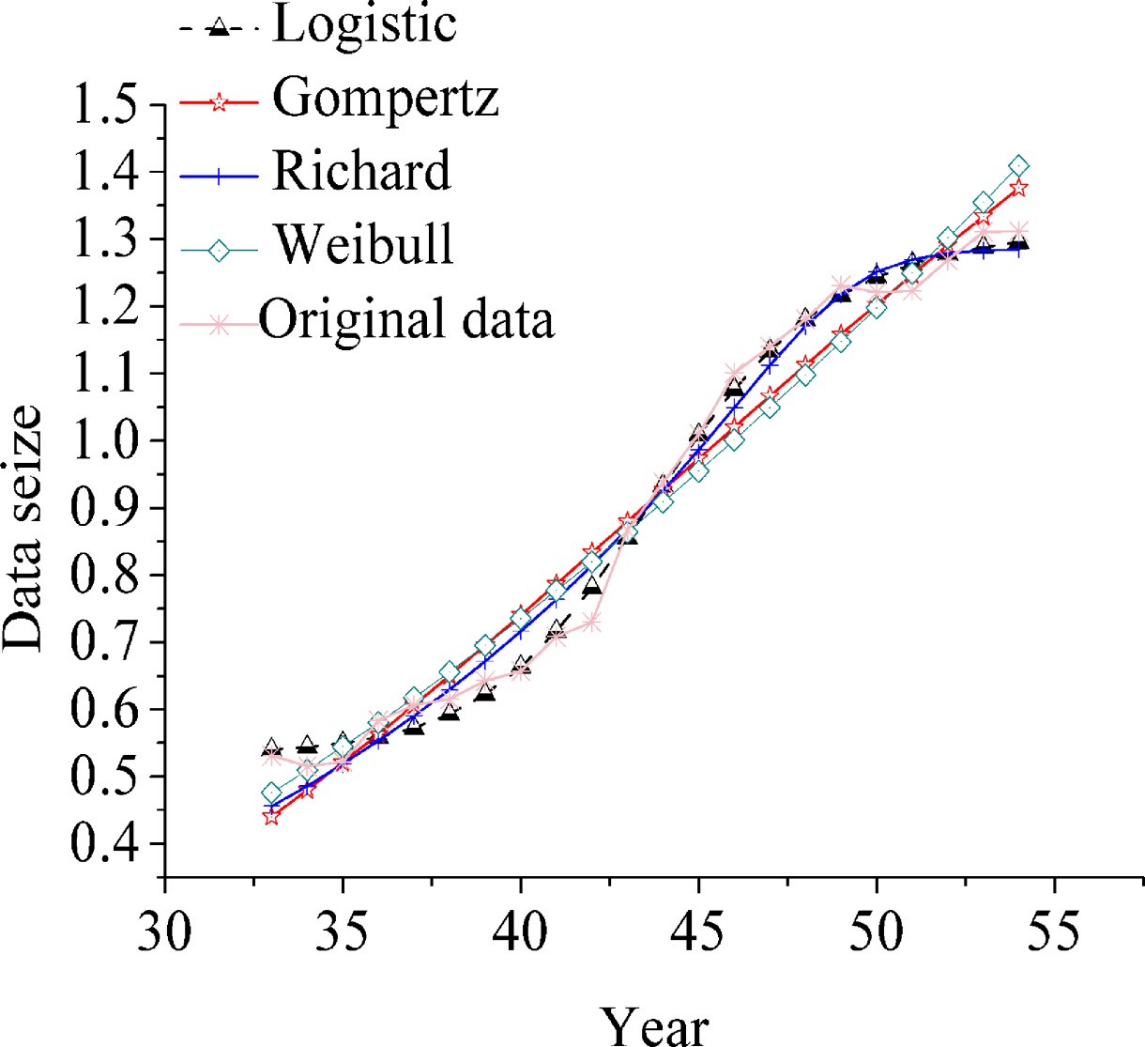
Comparison of four growth curve fitting curves in three life cycle (the second life cycle).

**Fig 9:**
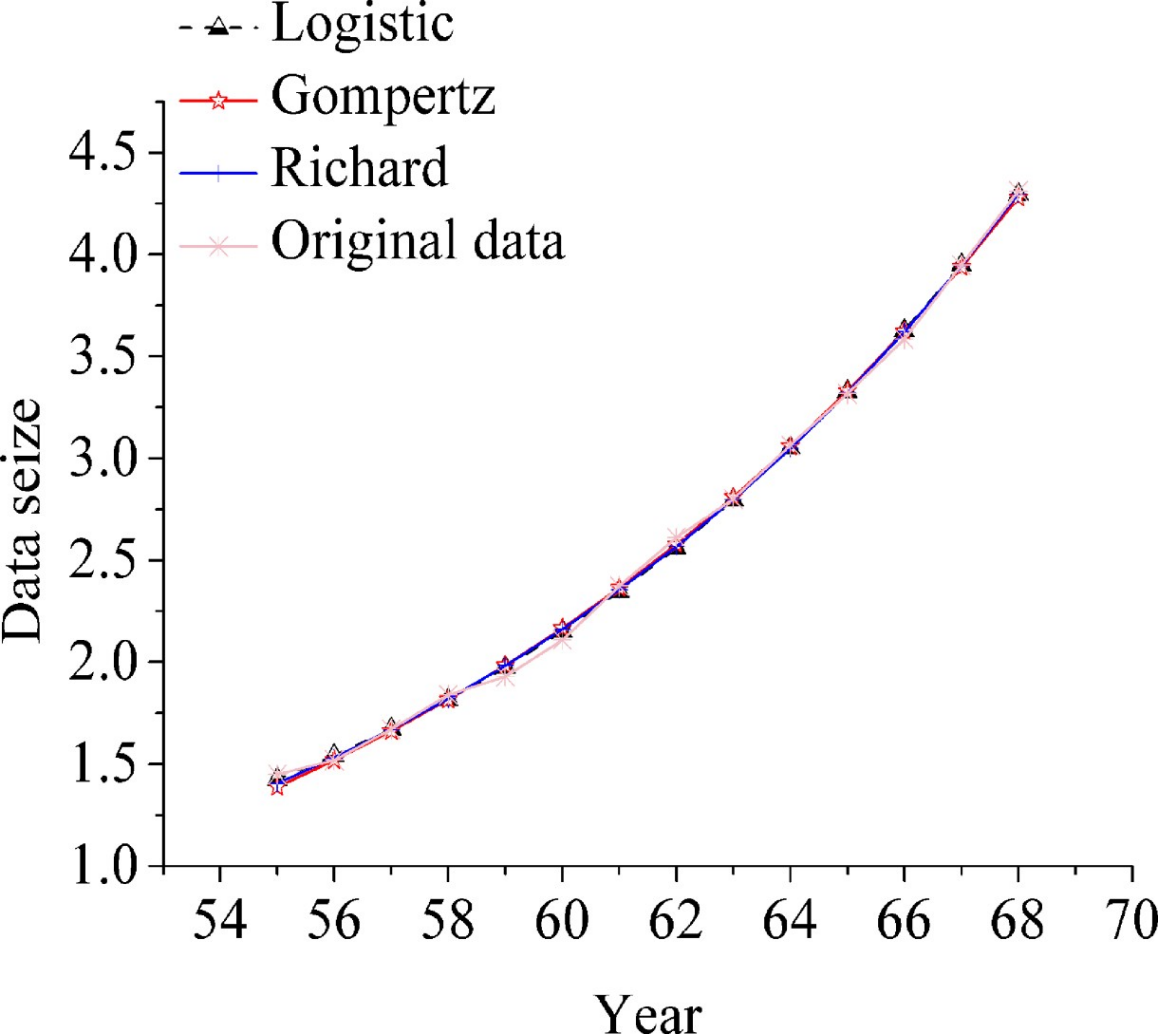
Comparison of four growth curve fitting curves in three life cycle (the third life cycle).

Four growth curve models are used to fit the life interval of the evolution and development of civil aviation transportation in China. The fitting results are listed in Table 5. In the first life cycle (1950–1981), the goodness-of-fit ranking of the four models is Gompertz > Weibull > Richard > logistic; in the second life cycle (1982–2003), the goodness-of-fit ranking of the four models is logistic > Richard > Gompertz > Weibull; in the third life cycle (2004–2017), the goodness-of-of-of-fit ranking of the other three models is greater than Weibull fitting divergence. The minor order is logistic > Richard > Gompertz. Logistic growth curve model accords with the growth characteristics of each life cycle of the development and evolution of civil aviation transportation in China. The fitting results are shown in Fig 7, Fig 8 and Fig 9.

**Table 5:** Fitting and residual analysis of four growth curves.

Year	Model	Goodness of fit	Minimum residual	Maximum residual
1950–1981	logistic	0.94513	-0.05013	0.00029
	Richard	0.94541	-0.05017	0.11261
	Weibull	0.94555	-0.05011	0.11162
	Gompertz	0.94729	-0.05016	0.11256
1982–2003	logistic	0.99314	-0.0514	0.03375
	Richard	0.98039	-0.08443	0.07474
	Weibull	0.95212	-0.09705	0.09949
	Gompertz	0.96019	-0.10316	0.09032
2004–2017	logistic	0.99868	-0.04586	0.05114
	Richard	0.99857	-0.05404	0.04521
	Weibull	×	×	×
	Gompertz	0.99841	-0.05836	0.06155

Note: "×" represents the divergence of fit.

### 5.2 Parameter estimation

The logistic growth curve model used in this study is a nonlinear function model. To determine the parameters of the growth curve model in each life cycle, the general method is to linearize the nonlinear function and then use linear function equation to fit the curve to determine the parameters. Logistic growth curve is one of them, which can be used to describe social and economic growth or the growth and reproduction speed of animals and plants. The growth curve’s image is a single peak curve, which indicates that the growth or growth process of logistic curve is slow-fast-slow i.e., three stages. By calculating the first derivative $\frac{dy}{dt}$ of logistic growth or growth rate function and making it equal to 0, the peak point *t*_*m*_ of speed can be obtained. It can be seen that when *t* = *t*_*m*_, the social and economic growth or the growth, development and reproduction of animals and plants are the fastest, reaching the peak period.

Finding the second derivative $\frac{d^{2}y}{dt^{2}}$ of logistic growth rate function, making it equal to 0, and getting *t*_1_,*t*_2_ make $\frac{d^{2}y}{dt^{2}}=0$. *t*_1_,*t*_2_ are the two inflexion points of velocity function, plus the peak point *t*_*m*_; therefore, the logistic function curve has three key feature points. These three points correspond to three key points in the process of social and economic growth or the growth, development and reproduction of animals and plants: the beginning, the peak and the end. Two inflection points *t*_1_ and *t*_2_ of velocity function can also be used to divide the growth process of logistic curve into gradual increase period (t = 0~*t*_1_), rapid increase period (t = *t*_1_~*t*_2_), and slow increase period (t = *t*_2_~∞).

Generally, the least square method is used to calculate the standard deviation between the prediction model and the actual model. In this study, the growth curve parameters of each life cycle are calculated by Origin 8.0 software. The results are presented in Table 6.

**Table 6:** Parameter estimation of logistic model over three life cycles.

Life cycle	Parameter	Results	Standard deviation	Goodness of fit	Formula of logistic model	Value interval of characteristic Point *t*
1950 ↓ 1981	A_1_	0.0833	0.0000	0.9469	$Y=0.7690-\frac{0.6857}{1+{\left(X/18.8121\right)}^{1.6667}},t\in [1,32]$	*t*_1_∈[2,3]; *t*_*m*_∈[7,8]; *t*_2_∈[15,16]
	A_2_	0.7690	0.1371			
	X_0_	18.8121	4.9969			
	P	1.6667	6.3046			
1982 ↓ 2003	A_1_	0.5157	0.0000	0.9930	$Y=1.3294-\frac{0.8137}{1+{\left(X/43.8379\right)}^{15.9963}},t\in [33,54]$	*t*_1_∈[40,41]; *t*_*m*_∈[43,44]; *t*_2_∈[47,48]
	A_2_	1.3294	0.0195			
	X_0_	43.8379	0.2246			
	P	15.9963	0.9916			
2004 ↓ 2017	A_1_	1.4501	0.0000	0.9926	$Y=5.5015-\frac{4.0514}{1+{\left(X/65.3306\right)}^{19.5610}},t\in [55,68]$	*t*_1_∈[65,66]; *t*_*m*_∈[71,72]; *t*_2_∈[78,79]
	A_2_	5.5015	0.4724			
	X_0_	65.3306	0.8533			
	P	19.5610	1.7153			

The parameters A_1_ in Table 6 represent the initial operating parameters in each life cycle; A_2_ represents the maximum operating parameters that can be reached in each life cycle; X_0_ represents the inflection point concentration of the evolution curve; and P represents the rate of evolution of civil aviation transport in each life cycle.

Through calculation and analysis, the curve velocity peak *t*_*m*_ of the first two life cycles is located between 1956 and 1957 and 1992 and 1993, respectively, and each life cycle has three characteristic features. The gradual increase in the first life cycle is between 1950 and 1952, the fast growth period is approximately 1952–1965, and the slow growth period is approximately 1965–1981. The gradual increase in the second life cycle is approximately 1981–1990, the fast growth period is approximately 1990–1997, and the slow growth period is approximately 1997–2003.

According to the analysis of the third life cycle, we can obtain the first inflection point *t*_1_∈[65,66] of the velocity function according to the data provided, and then according to the growth trend of the data, we can observe that the peak point *t*_*m*_ of the curve is between 2020 and 2021. Because the growth curve has the characteristics of "S" shape and central symmetry, the second inflection point *t*_2_∈[78,79] of the third life cycle can be obtained symmetrically. In other words, the gradual growth period of the third life cycle is approximately 2003–2015, the fast growth period is approximately 2015–2026, and the slow growth period is expected to start in 2026.

According to the above contents, we can see that the period of several major institutional reforms in the development of civil aviation transport in China is: the first stage (before 1980), the second stage (1980–1987), the third stage (1987–2002), the fourth stage (2002–2007), and the fifth stage (2008-). The three life cycle periods obtained by our method are: the first life cycle—(1850–1981), the second life cycle—(1982–2003), and the third life cycle—(2004-). Through comparison, we can find that the end time of the first life cycle coincides with the period of institutional reform in 1980, and the end time of the second life cycle coincides with the period of reform in 2002, which can prove that the life cycle we divide is reasonable from the perspective of institutional reform. It can be said that the development and evolution of China's civil aviation is driven by many factors, forming distinct characteristics of different stages of development.

## 6 Conclusions

1) Through the analysis of the evolution data and the growth curve theory, it is concluded that the growth curve model of CAAC conforms to the life cycle form of continuous growth curve. The evolution process of CAAC development can be analyzed by using the growth curve theory.

2) According to the maximum subordinate principle of adjusted goodness-of-fit, the development and evolution process of civil aviation in China from 1949 to 2017 can be divided into three life cycles; the first life cycle stage from 1950 to 1981, the second e from 1982 to 2003, and the third from 2004 to 2017.

3) The life cycle process of the development and evolution of civil aviation in China conforms to the growth law of the logistic model. According to the analysis of the parameters in the life cycle, it can be seen that there are three characteristic points in each life cycle, which correspond to the beginning, peak and end of each life cycle growth curve in turn. According to the inflection point of the growth curve velocity function, each life cycle can be divided into three stages, namely, gradual growth period, rapid growth period and slow growth period. According to the parameters of the third life cycle, we can see that China's civil aviation transportation is in the rapid growth period of the third life cycle. It is expected to reach maturity in 2026, and then begin to grow slowly.

4) There are many methods to select the data indicators. How to select the representative data indicators more scientifically is worth continuing to study, and whether there will be a more consistent growth model evolution in the selection of growth curve model is worth continuing to study.

5) The development and evolution of civil aviation in China has been pushed and pulled several times through time, technology and demand, forming a significant watershed stage of development. There are different stages of development in different stages. These characteristics of development have a distinct epoch mark. What is the driving force behind these developments? How to promote development will be worthy of further study by scholars.

## Supporting information

S1 File Data and analytical value of civil aviation development indicators in China. (XLSX)
